# The effect of temperature and humidity on adhesion of a gecko-inspired adhesive: implications for the natural system

**DOI:** 10.1038/srep30936

**Published:** 2016-08-02

**Authors:** Alyssa Y. Stark, Mena R. Klittich, Metin Sitti, Peter H. Niewiarowski, Ali Dhinojwala

**Affiliations:** 1 Integrated Bioscience Program, The University of Akron, Akron OH USA; 2 Department of Polymer Science, The University of Akron, Akron OH, USA; 3 Mechanical Engineering, Carnegie Mellon University, Pittsburgh PA, USA; 4 Max-Planck Institute for Intelligent Systems, Stuttgart, Germany

## Abstract

The adhesive system of geckos has inspired hundreds of synthetic adhesives. While this system has been used relentlessly as a source of inspiration, less work has been done in reverse, where synthetics are used to test questions and hypotheses about the natural system. Here we take such an approach. We tested shear adhesion of a mushroom-tipped synthetic gecko adhesive under conditions that produced perplexing results in the natural adhesive system. Synthetic samples were tested at two temperatures (12 °C and 32 °C) and four different humidity levels (30%, 55%, 70%, and 80% RH). Surprisingly, adhesive performance of the synthetic samples matched that of living geckos, suggesting that uncontrolled parameters in the natural system, such as surface chemistry and material changes, may not be as influential in whole-animal performance as previously thought. There was one difference, however, when comparing natural and synthetic adhesive performance. At 12 °C and 80% RH, adhesion of the synthetic structures was lower than expected based on the natural system’s performance. Our approach highlights a unique opportunity for both biologists and material scientists, where new questions and hypotheses can be fueled by joint comparisons of the natural and synthetic systems, ultimately improving knowledge of both.

The gecko adhesive system has received considerable interest due to its bio-inspired design applications. Principally known for strong, reversible, and dry adhesion^1^,^2^, geckos have become a focal species for synthetic design. Geckos use small microscopic hair-like structures (setae) on the bottom of their toes to provide close contact with the substrate they cling to^3^,^4^,^5^. It is these microscopic projections that have inspired hundreds of synthetic micro/nanostructures, which range from polymer pillars to carbon nanotubes and other hierarchically patterned structures^6^,^7^,^8^,^9^,^10^,^11^,^12^,^13^,^14^,^15^,^16^,^17^. Yet despite great enthusiasm towards building a better synthetic adhesive, much work has to be done on the natural system itself. For instance, somewhat surprisingly, we do not know the full composition or material organization of the setae, the very structures most often mimicked by these bio-inspired designs (see Jain *et al*.^18^ for a setal structure hypothesis). Furthermore, new properties of the natural system, such as adhesion to wet substrates and self-drying, are still being discovered and clarified^19^,^20^,^21^,^22^,^23^,^24^. Finally, although the mechanism behind gecko adhesion has been defined as mainly van der Waals-based^25^, the roles of capillary adhesion, electrostatic charging and material charge, and nanobubbles in the presence of water^20^,^26^,^27^,^28^,^29^,^30^, has called our attention to adhesion under the non-ideal conditions that geckos encounter on a routine basis.

One major challenge to fully resolving how geckos stick is also the most obvious: geckos are biological organisms and thus have inherent variability and complexity. Specifically, the biological adhesive system is likely adapted to work in multiple contexts and under a variety of circumstances, which may in turn mean that the system is not optimized to work maximally in any one condition. This versatility can be a useful property for synthetic adhesives, but can also be a central problem when designing tests to better elucidate the properties of the natural adhesive system. Take the case of capillary adhesion. The role of capillary adhesion has surfaced in the literature multiple times. In 2002, Autumn and colleagues dispelled the idea that capillary adhesion could be playing a significant role in gecko adhesion by showing that geckos were able to stick to both hydrophobic and hydrophilic substrates equally well during shear sliding^25^. In 2005, however, two separate studies found that the normal pull-off force of a single flattened pad (spatula), which forms the tips of the setae, was dependent on humidity, and thus on capillary adhesion in some form^27^,^30^. Up until this point, no groups had directly tested the effect of humidity on live animals. In response, Niewiarowski *et al*. tested whole animal shear adhesion of live geckos at two temperatures and a series of humidity levels^31^. Supporting the work on gecko spatulae, they found that adhesion was humidity dependent but, surprisingly, this was only the case at low temperature^31^. At high temperature humidity had no affect on adhesion. Further investigation of humidity effects on setal adhesion showed that normal adhesive force at 100% RH increased as load increased^32^, although very high loads and high sliding velocity negated this improvement^33^. The former study proposed that one mechanism for enhanced adhesion may be rearrangement of surface chemical groups in the presence of water^32^. Surface group rearrangement was later confirmed through surface sensitive techniques^34^. Finally, the dominant hypothesis explaining the increase in adhesion in the presence of high humidity (>80% RH) is that setal modulus is reduced and thus increases contact area and adhesive performance^35^,^36^. Recently capillary adhesion has again come up in the context of adhesion to wet substrates, where small nanobubbles may cause capillary bridging, allowing for the underwater adhesion experimentally measured in whole animal studies^20^,^21^. Currently however, there are no hypotheses which can sufficiently explain why adhesion of live geckos depends both on humidity *and* temperature, and this debate has been relatively quiet for years.

Using the capillary adhesion example, it is quite clear that the context in which adhesion occurs is critical. Load, sliding velocity, level of humidity, temperature, presence of water, dynamic surface groups, and other parameters can impact adhesion. Furthermore, whole animal studies are consistently different than those testing single spatula, setae, and setal arrays^1^, making it difficult to compare across studies. Thus, a challenging question arises: how do we separate all of these dynamic and complex components of the natural gecko adhesive system to decipher the system’s intrinsic fundamental properties? We propose a non-traditional approach to answer this question. Instead of testing the natural system, we take advantage of the controlled parameters of a previously designed gecko-inspired synthetic to probe the basic properties innate to the natural system. By using this approach, factors such as modulus change and surface group reorganization can be controlled or eliminated, allowing us to directly compare and contrast synthetic trials with the previous whole animal results. While this approach is not novel^37^,^38^,^39^,^40^,^41^, we believe it is highly underutilized, particularly in the field of gecko adhesion.

In this study, we tested shear adhesion of a high performing mushroom-tipped gecko-inspired synthetic and used it to probe the effect of humidity and temperature on a dry fibrillar adhesive. We replicated the conditions Niewiarowski *et al*. used for whole animal experiments, which consisted of measuring maximum shear adhesion of live geckos on a glass plate at two temperatures (12 °C and 32 °C) and four different humidity levels (30%, 55%, 70% and 80% RH)^31^. Our aim for this comparison was to probe the complex interaction between temperature and humidity on adhesion at the whole animal scale. We believe that fundamental adhesion properties related to humidity and temperature will be replicated in this test, and that differences between the natural adhesive system and the synthetic will help to direct future work on the natural adhesive system. Thus, this work not only tests the performance of synthetic mimics which can adhere at varying temperature and humidity jointly, but is also a valuable source of information for future experiments of the natural system, pushing the field forward and in directions we may not have been able to identify without this controlled comparative design.

## Results

### Maximum Shear Adhesion of Gecko-inspired Synthetic Microfibers

**Figure 1:**
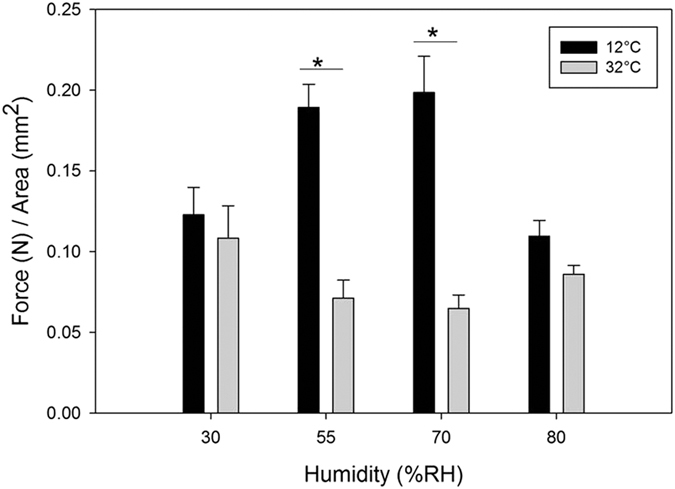
Average force per unit area of synthetic gecko tape. Samples were tested in two temperatures (12 °C black bars; 32 °C grey bars) at four different humidity settings. Bars with an asterisk above signify a statistically significance difference between the two groups (p < 0.05). Error is reported as mean ± 1 s.e.m.

There was a significant effect of temperature and humidity on shear adhesion of the gecko-inspired synthetic microfibers (F_8,39_ = 11.47, p < 0.0001; Table 1). When investigating all pairs, we found that at low temperatures (12 °C), samples tested at 70% and 55% RH did not differ from one another and were significantly higher than samples tested at these same humidity levels at 32 °C. Samples tested at 30% RH and 80% RH did not differ from one another in each temperature group (Fig. 1).

### Adhesion Behavior of Gecko-inspired Synthetic Microfibers

**Figure 2:**
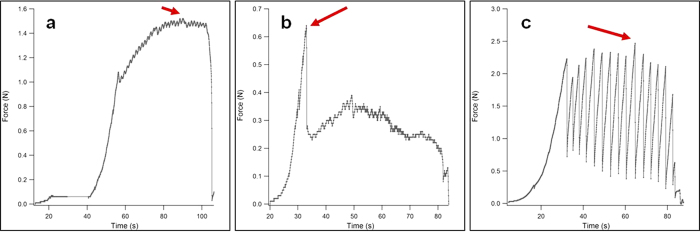
Typical force profiles of each behavior observed during experimental trials. These behaviors were characterized as a “U” shaped curve (**a**), a “V” shaped curve (**b**), and a “Stick-Slip” curve (**c**). Arrows denote maximum shear adhesion values where maximum shear adhesion is reached during sliding (**a,c**) or when maximum shear adhesion is reached first, then sliding occurs (**b**).

We defined three adhesion behavior types from the experimental force profiles. First, some force profiles exhibited a “U” shape (U), where maximum adhesion occurred at some point in a smooth “U” shaped curve. This profile suggests that sliding friction is greater than static friction. Second, some force profiles exhibited a “V” shape (V), where maximum adhesion occurred at the apex of the “V”. This profile implies static friction is stronger than sliding friction. Finally, the third type of force profile was “Stick-Slip” (SS), where maximum adhesion occurred during periodic stick-slip events. In this profile static and sliding fiction are intermittent and dynamic, where in periods of stick, static friction is dominant, and in periods of slip, sliding friction is dominant. Some samples showed multiple behaviors in one test, thus behavior was assigned based on where the maximum force value occurred. Examples of all three force profiles are shown in Fig. 2. We found that there was no difference in force profile behavior when temperature and humidity were considered jointly, though this was marginal (Person χ^2^ = 22.05, p = 0.0775). When investigating temperature and humidity independently, we found that the two temperatures tested had significantly different behavior (Person χ^2^ = 8.35, p = 0.0154), where samples tested at 32 °C had all three behavior types (U, V, and SS) and those tested at 12 °C only had two (SS and V). Humidity had no significant effect on adhesion behavior when considered independently (Person χ^2^ = 8.05, p = 0.2348).

## Discussion

Our work represents one of very few examples using a gecko-inspired synthetic microfiber array to probe questions about the natural gecko adhesive system. Results from both the natural system and the synthetic system are strikingly similar: adhesion only increases with increasing humidity at cool temperatures. In the biological system several hypotheses have been proposed, but not yet resolved, for the effect of humidity on adhesion. First, the reduction of setal modulus increases available contact area by allowing the setae to become more compliant and flexible, thus adhesion increases at high humidity (>80% RH). Second, surface restructuring causes an increase in adhesion in the presence of water (i.e., deposited surface water layers due to high humidity). Finally, non-independent from the change in modulus hypothesis, material swelling may occur (in addition to material softening), causing an increase in contact area and therefore adhesion. Unfortunately, none of these hypotheses can be completely independently tested in the natural system (but see Badge *et al*.^19^ for an example of controlled surface chemistry). In this study however, we found that the synthetic system can be used as a good proxy for the natural, thus we can use the synthetic system to directly test these three hypotheses to elucidate the mechanism behind the natural system’s behavior.

The first, and most dominant hypothesis, is the reduction of setal modulus in high humidity (>80%)^35^,^36^,^42^,^43^. To investigate the interaction of temperature and humidity on the modulus of synthetic samples, we measured the modulus of plain sheets of the synthetic material (550 μm thick) under four of the eight test conditions (12 °C and 32 °C at ~40% RH and 80% RH). Tensile tests were done following ASTM D638-10. The samples were placed in the controlled environment for 1 hour prior to testing. A minimum of 4 samples per condition were tested using a 210 Universal Test Machine (TestResources, Inc, Shakopee, MN) at the rate of 5 mm/min. We found that there was a significant difference in modulus (Analysis of Variance (ANOVA), F_3,22_ = 7.00, p = 0.0018), where temperature was the only significant effect driving this difference (p = 0.0007). Modulus at the 12 °C temperature set point was significantly higher than the modulus measured at 32 °C (2.97 ± 0.20 MPa at 12 °C; 1.87 ± 0.14 MPa at 32 °C). This means that overall, temperature influences the synthetic modulus but humidity does not, suggesting that the temperature by humidity interaction observed in adhesion tests of the synthetic is not driven by changes in modulus. Furthermore, given the increase in modulus at 12 °C, we would expect the opposite adhesion results, where shear adhesion would reduce at cold temperatures rather than improve. It is important to note the modulus of gecko setae is higher than the synthetic (~2 GPa)^44^ and that modulus may decrease on the surface of the polymer pillars (<40 nm), as discovered in thin films^45^. Interestingly, despite differences in bulk modulus, potential surface-layer changes and material changes of the synthetic due to temperature alone, shear adhesion behavior of the synthetic was similar to that of the animal across the ranges of temperature and humidity tested (with the exception of 12 °C and 80% RH).

Similar to the first hypothesis, we expected the second and third hypotheses (surface group restructuring and swelling) to have no major effect on adhesive performance of the synthetic. To test this, we again utilized the synthetic and investigated changes in contact angle and weight in high humidity to confirm that the synthetic material was stable during adhesion tests. To test the second hypothesis, we measured the contact angle of water on the smooth backing material in four of the eight treatments used in adhesion tests (12 °C and 32 °C at ~35% RH and 80% RH). Contact angle was averaged over three measurements per sample using ImageJ to analyze images from a custom goniometer. At least 10 samples were used per treatment and all were held in the test environment for at least 1 hour. We found no significant change in water contact angle across the treatments (ANOVA, F_3,45_ = 0.35, p = 0.7929), suggesting that no significant surface restructuring occurred in the synthetic adhesive. Contact angle for a smooth sheet of the fiber material yields a contact angle of 78.5 ± 1.4° at room temperature and humidity, which is within the range of what is expected for the natural system based on measurements and theoretical calculations (70–90°)^19^,^46^. To test the final hypothesis, we used a Cahn 25 Automatic Electrobalance under 85% RH conditions and measured change in mass over a 2 hour period. We found only a 0.23 ± 0.05% increase in mass in four samples tested. This is in contrast to β-keratin, one of the primary material components of gecko setae. Taylor *et al*.^47^ observed a 2.4-fold difference in wet mass of bird feather β-keratin between 50% and 100% RH. Despite the clear differences in the synthetic and natural systems with regards to surface restructuring and swelling, our results with the synthetic show that it is unlikely these two properties of the natural system contribute to adhesive performance in variable temperature and humidity environments (though see our discussion below on adhesion at 12 °C and 80% RH).

Because it is unlikely that a reduction of modulus, surface group restructuring, or material swelling related to the interaction of temperature and humidity occurs to a significant degree in the synthetic system under our experimental conditions, we suggest these mechanisms are not important in explaining variation in the natural system under the same conditions (but see the discussion below on where the two systems differ). One alternative may be to reconsider the influence of adsorbed water on shear adhesion. Water can influence shear adhesion by two main mechanisms. First, adsorbed water layers may lubricate the contact interface and reduce shear adhesion and friction. Secondly, capillary bridges may form between the contact elements and the substrate and increase normal adhesion. Both mechanisms can change as humidity increases, however, why increased shear adhesion of the natural and synthetic systems occurs only at cool temperatures is not explained completely by either mechanism. In whole animal experiments surface water on a hydrophilic glass surface, like the one used here, produced significantly reduced adhesion at room temperature^21^. Interestingly, thin water layers on a hydrophilic sapphire prism were successfully expelled by the gecko toe at room temperature^34^. Similar findings, including water pockets between contacting surfaces, air pockets between hierarchal structures, and lubricating layers of water have been determined in synthetic materials and other biological adhesive systems^48^,^49^,^50^,^51^. Thus, it is possible that a combination of capillary adhesion and expulsion of water from the contact interface is controlling the trends observed in this study. Observationally, we noticed that at low temperatures, strong stick-slip or stick events dominated adhesion behavior of the synthetic system. This supports our hypothesis that the pillars are making dry contact in the cool temperature but not in the warm. It is important to keep in mind however, that synthetic samples showed no difference in adhesion behavior related to humidity, suggesting that temperature, and perhaps the reduction of modulus of the synthetic material at 32 °C, drives our observation of stick-slip or stick events, rather than water layers. We suggest a detailed investigation of the thickness of water layers deposited on the surface of glass at varying temperature and humidity regimes to confirm our water layer hypothesis, and a more thorough investigation of the temperature effect on material properties of the pillars to determine the source of the adhesion behavior profiles.

While there are startling similarities between the natural and the synthetic systems, is also important to investigate where these two systems differ. One clear difference between the synthetic and natural system adhesion results is at 12 °C and 80% RH. In the natural system, adhesion was almost twice as high at 12 °C and 80% RH as compared to the low humidity trials at 12 °C and 35% RH^31^. Interestingly, this was not the case in the synthetic system, where there was no difference between these two treatment groups (Student’s t-test, t = 0.93, df = 9.5, p = 0.3736). The difference in whole animal adhesion and synthetic adhesion suggests that the two systems behave differently in this particular environment. Keeping in mind the dynamics of the natural system, it is possible that in this condition the natural system changes to maintain adhesion in ways the synthetic system cannot. This includes surface restructuring (i.e., change from methyl to methylene^34^), decrease in setal modulus at >80% RH, swelling, or viscous dissipation^35^. The discrepancy at this particular environmental condition is interesting and has produced several specific hypotheses that can be tested in the future.

Finally, this experiment also highlights the ability of a synthetic gecko-inspired mushroom-tipped adhesive to adhere in a variety of environmental conditions. Specifically, while temperature effects on pressure sensitive adhesives (PSAs) have been investigated previously^52^, this study focuses on the joint effect of two common environmental parameters (i.e., temperature and humidity). For instance, high humidity can be a challenge for traditional PSAs that struggle to adhere in the presence of a surface water layer^53^,^54^, but how this effect is further compounded by temperature is unknown. In these experiments, we see that adhesion of the synthetic at 80% RH and 30% RH does not differ across either temperature range, and can even be improved at intermediate humidity levels and cool temperature. In addition, the resilience of this gecko-inspired synthetic adhesive to shear adhesion was quite impressive. Unlike the natural system, high shear did not remove or detach pillars, and sample images taken prior to testing and after testing looked very similar. Despite this, we believe that the synthetic adhesive has specific room for improvement where it differs from the natural system at 12 °C and 80% RH. We believe that investigations of the natural system will help inform changes in design that can improve adhesion in this environmental condition and others.

In summary, by comparing the natural and synthetic systems’ performance and adhesion behavior, we are able to offer a new perspective on experiments focusing on gecko adhesion at the whole animal scale. First, we found that several of the prevailing hypotheses about how geckos stick in humid environments may not be viable. Our results once again call into question the roles of capillary adhesion and interfacial water layers in the gecko adhesive system. Secondly, by investigating the particular temperature and humidity regime where the systems differ, our understanding of the natural adhesive system will improve, as well as produce ideas for design parameters that can increase synthetic performance in conditions where the natural system has higher performance. Thirdly, the stability of this adhesive in challenging environmental conditions, such as high temperature and humidity, has several applications to real-world adhesive use and commercialization. Finally, we hope our work inspires further investigations into how synthetic designs can improve the type of questions we ask about the natural adhesive system, creating an “idea feedback loop” between the natural and synthetic system, both of which have been generally tested independently.

## Materials and Methods

A polyurethane elastomer mushroom-tipped synthetic gecko-inspired adhesive was obtained from Carnegie Mellon University. Samples were manufactured using methods similar to those outlined by Murphy *et al*.^55^. Base pillars were 100 μm high and 50 μm in diameter with enlarged mushroom tips. Six samples were tested per temperature and humidity condition. Samples were cut into squares with an average area of 19.1 ± 0.6 mm^2^. Each sample was imaged independently both before and after experimental trials to check for defects and experimental damage. Samples were attached to a glass slide with mushroom caps facing upward using a cyanoacrylate gel adhesive that was slightly cured prior to attachment, making sure the gel adhesive did not cover the sample. A controlled weight (174.5 g) was placed on the sample before a protective backing layer was removed from the pillar caps. This pre-weight was used to ensure that the samples had been fully pressed into the glue and were horizontally level. No sample was used more than once and samples that had premature loading (prior to testing or during testing in a non-controlled way) were discarded.

Each sample slide was placed in a test arena as described in Badge *et al*.^19^. Slides were attached to the bottom of the arena with double sided tape. A weighted (~46 g) glass slide was tethered via a nylon string to a force sensor mounted on a motorized track (see Badge *et al*. for a schematic). The weighted glass side was pulled across the sample in the shear direction for about 4 cm at a controlled rate. The glass slide was cleaned with ethanol and water after each sample and dried with a Kimwipe. Force was recorded over time and maximum force was determined from the force curve post-experiment. Adhesion behavior was also characterized post-experiment using the force curves. All experiments were conducted in an environmentally controlled walk-in chamber set to either 12 °C or 32 °C and all experiments were conducted within ±2 °C of this range. Humidity was controlled within ±2% of the following set points: 30%, 55%, 70% and 80% RH. Each of the four humidity set points were tested at the two temperature settings, totaling eight different treatments. All samples were equilibrated to the treatment temperature and humidity for at least 1 hour prior to experiments.

Samples were tested semi-randomly. The effect of temperature, humidity, and their interaction on shear adhesion was tested using an analysis of covariance (ANCOVA) where area was used as a covariate. Individual comparisons across groups were done using a Tukey HSD test to control for multiple tests. To compare adhesion behavior across temperature and humidity we used a Pearson Chi-square test. Means are reported mean ±1 s.e.m.

### Data availability

The raw data are provided in the supplementary information.

## Additional Information

**How to cite this article**: Stark, A. Y. *et al*. The effect of temperature and humidity on adhesion of a gecko-inspired adhesive: implications for the natural system. *Sci. Rep.* **6**, 30936; doi: 10.1038/srep30936 (2016).

## Supplementary Material

Supplementary Information

## Figures and Tables

**Table 1:** Analysis of covariance of the effect of temperature, humidity, and their interaction on shear adhesion of synthetic gecko tape.

Source	DF	SS	F Ratio	p-value
Temperature	1	19.3342	56.1254	<0.0001*
Humidity	3	1.0476	1.0137	0.3970
Temperature X Humidity	3	10.2127	9.8822	<0.0001*
Area	1	0.0048	0.0139	0.9066

Area is used as a covariate. Values marked with an asterisk signify statistical significance (p<0.05).
